# Prognostic Value of Coronary Flow Capacity by ^82^Rb PET in Patients With Suspected Coronary Artery Disease and Normal Myocardial Perfusion at Semiquantitative Imaging Analysis

**DOI:** 10.1161/CIRCIMAGING.124.016815

**Published:** 2024-11-07

**Authors:** Emilia Zampella, Roberta Assante, Adriana D’Antonio, Teresa Mannarino, Valeria Gaudieri, Carmela Nappi, Parthiban Arumugam, Mariarosaria Panico, Pietro Buongiorno, Mario Petretta, Alberto Cuocolo, Wanda Acampa

**Affiliations:** 1 Department of Advanced Biomedical Sciences, University Federico II, Naples, Italy (E.Z., R.A., A.D., T.M., V.G., C.N., P.B., A.C., W.A.).; 2 Department of Nuclear Medicine, Central Manchester Foundation Trust, Manchester, United Kingdom (P.A.).; 3 Institute of Biostructure and Bioimaging, National Research Council, Naples, Italy (M. Panico).; 4 Institute for Research and Healthcare SYNLAB SDN, Diagnostic Imaging, Naples, Italy (M. Petretta).

**Keywords:** coronary artery disease, heart ventricles, prognosis, myocardial perfusion imaging, positron emission tomography

## Abstract

**BACKGROUND:**

Coronary flow capacity (CFC) is a measure that integrates hyperemic myocardial blood flow and myocardial flow reserve to quantify the pathophysiological impact of coronary artery disease on vasodilator capacity. We assessed the prognostic value of CFC derived from ^82^Rb positron emission tomography/computed tomography in patients with suspected coronary artery disease and normal myocardial perfusion imaging.

**METHODS:**

We studied 1967 patients with suspected coronary artery disease and normal myocardial perfusion at the semiquantitative analysis of stress/rest cardiac ^82^Rb positron emission tomography/computed tomography imaging. Coronary artery calcium scores were calculated and categorized into 3 groups: 0, 0.1 to 99.9, and ≥100. Patients were classified as having myocardial steal, severely reduced CFC, moderately reduced CFC, mildly reduced CFC, minimally reduced CFC, or normal flow using previously defined thresholds. The outcome end points were myocardial infarction and cardiac death, whichever occurred first.

**RESULTS:**

During a mean time of 41±27 months, 49 events occurred (2.5% cumulative event rate, with an annualized event rate of 0.5% person-years). At multivariable Cox analysis, coronary artery calcium score categories and impaired CFC resulted as independent predictors of events (both *P*<0.001). The annualized event rate was higher in patients with impaired CFC compared with those with normal CFC (*P*<0.05). Kaplan-Meier analysis showed that patients with impaired CFC were at the highest risk of events.

**CONCLUSIONS:**

In patients with suspected coronary artery disease and normal myocardial perfusion, impaired CFC is associated with a higher risk of cardiac events. Evaluating CFC can help identify patients’ candidates for additional therapies to prevent future events.

**CLINICAL PERSPECTIVE**

In patients with suspected coronary artery disease, risk stratification is crucial to establish treatment strategies. Myocardial perfusion imaging by positron emission tomography provides an accurate evaluation of both atherosclerotic burden and vascular function by the absolute quantification of myocardial blood flow and myocardial flow reserve. These parameters showed a high prognostic value in several populations. However, myocardial flow reserve derives from the ratio between hyperemic and resting myocardial blood flow, and its estimation may be affected also under physiological resting conditions, resulting in discrepant findings compared with hyperemic blood flow. Coronary flow capacity (CFC), an index that integrates hyperemic myocardial blood flow and flow reserve, may be able to better risk stratify patients. The current study shows that in patients with suspected coronary artery disease, the presence of impaired CFC derived from ^82^Rb positron emission tomography perfusion imaging increases the risk of future cardiac events. Despite that the risk of events was higher in patients with reduced myocardial flow reserve and in those with impaired CFC, CFC seems to provide a better risk stratification than flow reserve alone. CFC also resulted as independent predictors of events, with a high stratification power. These findings support the need to carefully interpret both myocardial blood flow and flow reserve values to have an overall evaluation of coronary vascular function and to better identify patients at higher risk of events.

**See Editorial by Gould and Johnson**

Coronary artery disease (CAD) is a multifactorial process that may affect coronary vascular beds at different levels, involving both epicardial and microvascular compartments.^1^ Cardiovascular events may occur after a latent phase of clinically unapparent disease in which patients may have normal functional tests.^2^ Myocardial perfusion imaging by positron emission tomography (PET)/computed tomography (CT) is able to provide an accurate evaluation of both atherosclerotic burden and vascular function, through coronary artery calcium (CAC) score measurement and absolute quantification of myocardial blood flow (MBF) and myocardial flow reserve (MFR).^3^ MFR is the most validated index of coronary vascular function, able to provide an accurate evaluation of both epicardial and microvascular compartments.^4,5^ The prognostic value of MFR has been demonstrated in several populations, also in the absence of other myocardial perfusion and structural abnormalities.^6–10^ In most patients, hyperemic MBF and MFR provide concordant findings. However, MFR derives from the ratio between hyperemic and resting MBFs, and its estimation may be affected also under physiological resting conditions, resulting in discrepant findings compared with hyperemic MBF. Consequently, both parameters should be considered in the interpretation of myocardial perfusion imaging results.^11^ Coronary flow capacity (CFC) has been proposed as a comprehensive measure of the coronary vascular status, integrating both hyperemic MBF and MFR results into a color-coded scatterplot.^12,13^ CFC evaluation provides an objective, physiological quantification of CAD associated with high-risk events.^14–16^ Recently, the concept of modified CFC has been introduced by integrating the regional CFC category within each coronary territory into the entire CFC category for each patient.^17,18^ The prognostic value of this approach has been tested in a cohort of patients with and without evidence of CAD derived from ^15^O-H_2_O PET imaging.^18^ We assessed the prognostic value of CFC derived from ^82^Rb cardiac PET in predicting cardiac outcome in patients with no evidence of CAD and normal myocardial perfusion, defined as total perfusion defect <5% of the total left ventricle.

## Methods

### Data Availability Statement

The data that support the findings of this study are available from the corresponding author upon reasonable request.

### Study Population

We studied 4560 consecutive patients who underwent stress/rest ^82^Rb cardiac PET/CT imaging between January 2011 and December 2021. A total of 2026 patients were excluded for (1) documented history of CAD defined as luminal stenosis >50% at coronary angiography, previous percutaneous coronary intervention, coronary artery bypass graft surgery, or myocardial infarction and (2) uncontrolled atrial fibrillation, pacemaker, or prosthetic valve. Other 481 patients were also excluded for the presence of abnormal myocardial perfusion imaging, leaving 2053 subjects for the analysis. For each patient, demographic and clinical characteristics, including the presence of coronary risk factors, were noted. The study complies with the Declaration of Helsinki. The review committee of our institution approved this study, and all patients gave informed consent (Comitato Etico, Università Federico II; protocol number 110/17).

### PET/CT Imaging

The patients were asked to discontinue nitrates for 6 h, calcium channel blockers, caffeine-containing beverages for 24 hours, and β-blockers for 48 hours before PET/CT imaging. Rest and stress cardiac PET/CT images were acquired using Biograph mCT 64-slice scanners (Siemens Healthcare). After a CT scout to check patient position, a low-dose CT (0.4 mSv; 120 kVp; effective tube current, 26 mA [11-mAs quality reference]; 3.3 s) was performed for CAC score measurements and attenuation correction during normal breathing before and after PET acquisitions. For both rest and stress imaging, a 6-min list-mode PET acquisition was acquired after 1110 MBq of ^82^Rb was injected. For stress images, the pharmacological stress test was performed by adenosine administration (140 μg×kg^−1^×min^−1^ for 4.5 minutes, with tracer injection between 2 and 2.5 minutes). Both rest and stress dynamic images were reconstructed into 26-time frames (12×5, 6×10, 4×20, and 4×40 s) using the vendor standard ordered-subset expectation maximization 3-dimensional reconstruction (2 iterations, 24 subsets) with 6.5-mm Gaussian postprocessing filter. The images were corrected for attenuation using the low-dose CT. Hemodynamic parameters and 12-lead ECG were recorded at baseline and throughout the infusion of adenosine.

### Calcium Scoring

For CAC scoring, the rest CT axial reconstructions were transferred to a dedicated workstation (Vitrea Workstation, Toshiba Medical Systems, Tokyo, Japan) for postprocessing and subsequent analysis. Coronary calcification was defined as a plaque with an area of 1.03 mm^2^ and a density ≥130 HU. CAC scores by Agatston were calculated according to the method described^3^ and categorized into 3 groups: 0, 0.1 to 99.9, and ≥100. Experienced nuclear medicine physicians analyzed the CT studies blinded to the PET results.

### Imaging Analysis

**Figure 1.**
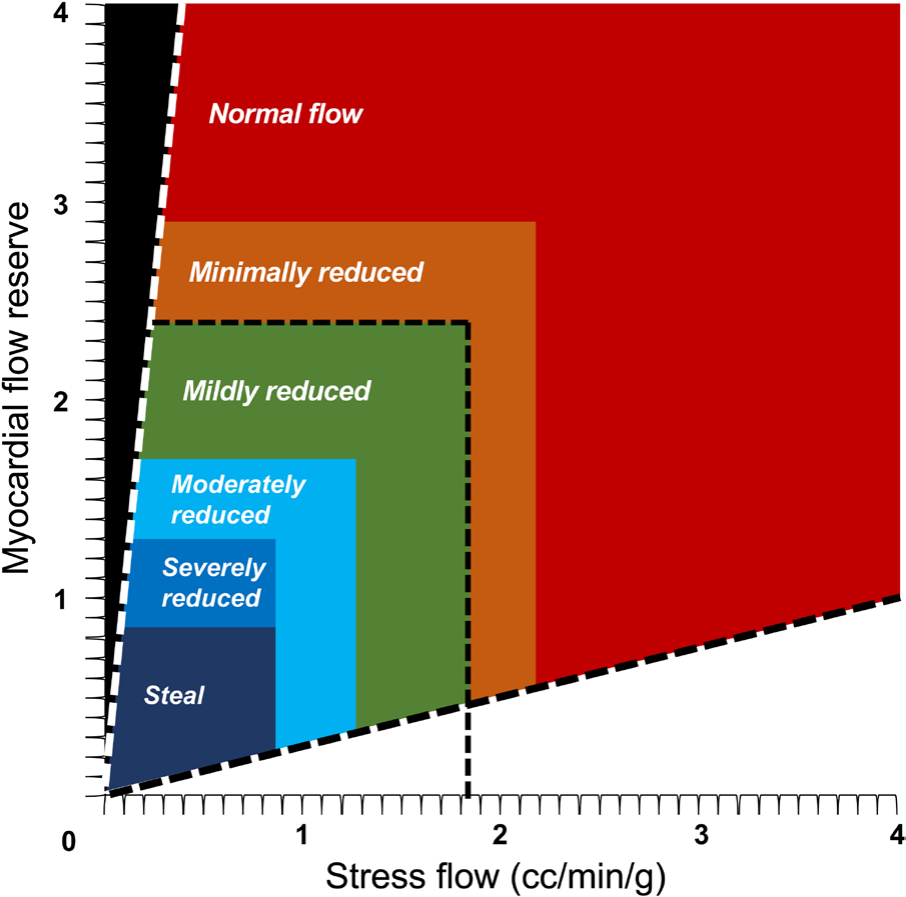
**Coronary flow capacity (CFC) map according to myocardial flow reserve and stress flow.** CFC was classified as normal flow, minimally reduced flow, mildly reduced flow, moderately reduced flow, severely reduced flow, and myocardial steal. CFC categories were defined as preserved in the presence of normal or minimally reduced flow or impaired in the presence of mildly reduced or more reduced flow. Reproduced with permission of Elsevier from Johnson and Gould.^13^

Trans-axial PET perfusion images were automatically reoriented into short-axis and vertical and horizontal long-axis slices. Myocardial perfusion was assessed using standardized segmentation of 17 myocardial regions using automated software (Cedars-Sinai Medical Center, Los Angeles, CA).^19^ The total perfusion defect was considered normal when <5% of the total left ventricle.^20^ MBF was calculated (mL/min per g) for globally and for each vascular territory from the dynamic rest and stress imaging series with commercially available software (FlowQuant, University of Ottawa Heart Institute).^21^ From the ratio of hyperemic to baseline MBF, MFR was calculated and considered reduced when <2. CFC was measured according to regional MBF and MFR previously proposed thresholds^13,14^ and classified as normal flow, minimally reduced flow, mildly reduced flow, moderately reduced flow, severely reduced flow, and myocardial steal (Figure 1). CFC categories were defined as preserved in the presence of normal or minimally reduced flow or impaired in the presence of mildly reduced or more reduced flow.^17,18^

### Outcomes

Follow-up was obtained by using a questionnaire that was assessed by a phone call to all patients or referring physicians and by review of hospital or physicians’ records. The outcome end points considered were myocardial infarction and cardiac death, whichever occurred first. The cause of death was confirmed by a review of the death certificate, hospital chart, or physician’s records. Death was considered of cardiac origin if the primary cause was defined as acute myocardial infarction, congestive heart failure, valvular heart disease, sudden cardiac death, and cardiac interventional/surgical procedure-related. Myocardial infarction was defined when >2 of the following 3 criteria were met: chest pain or equivalent symptom complex, positive cardiac biomarkers, or typical electrocardiographic changes.^22^ The date of the last examination or consultation was used to determine the length of follow-up.

### Statistical Analysis

Continuous data were expressed as median (interquartile range) for the nonnormally distributed continuous variables and compared using the Mann-Whitney *U* test. Categorical variables were expressed as numbers (%) and compared for the differences by the χ^2^ and Fisher exact test as appropriate. *P*<0.05 (2-sided) was considered statistically significant. To evaluate the effects of CFC categories on outcome, an omnibus χ^2^ test was performed, and if significant, post hoc multiple pairwise comparisons with Bonferroni correction were tested. The annualized event rate (AER), expressed as % person-years, was calculated as the cumulative number of events divided by person-time. Hazard ratios with 95% CIs were calculated by univariable and multivariable Cox regression analyses. Variables showing *P*<0.05 at univariable analysis were considered for multivariable analysis. The additional value of variables added sequentially was evaluated on the basis of the increases in the overall likelihood ratio statistic. Five different models were considered: model 1, including clinical data and CAC score; model 2, clinical data, CAC score, and global MFR; model 3, clinical data, CAC score, and global and regional MFR; model 4, clinical data, CAC score, global and regional MFR, and global CFC; and model 5, clinical data, CAC score, global and regional MFR, and global and regional CFC. Event-free survival curves were obtained by the Kaplan-Meier method and compared using the log-rank test. Classification and regression tree (CART) analysis was performed using the CART Stata package for failure time data.^20^ For this analysis, only variables showing *P*<0.05 at multivariable analysis were considered. Statistical analysis was performed with Stata 18 software (StataCorp, College Station, TX).

## Results

Follow-up was available in 1967 (96%) patients. During a mean time of 41±27 months, 49 events occurred (2.5% cumulative event rate, with an AER of 0.5% person-years). The events were myocardial infarction in 33 (67%) patients and cardiac death in 16 (33%) patients. Clinical characteristics and imaging findings according to events are reported in Table 1. Patients with events had a higher prevalence of male sex, diabetes, and hypertension compared with those without events. Patients with events also showed a minor prevalence of CAC score 0, lower hyperemic MBF and MFR values, and a higher prevalence of impaired MFR and CFC. The CFC categories showed a significant effect on outcome (χ^2^ of 34 for global and χ^2^ of 50 for regional CFC; both *P*<0.001). For global CFC, post hoc comparison demonstrated a significant difference for mildly reduced subgroups versus normal and minimally reduced subgroups (*P*<0.05). For regional CFC, there was a significant difference between mildly mild and moderately reduced subgroups versus normal and minimally reduced subgroups (both *P*<0.05).

**Table 1.**
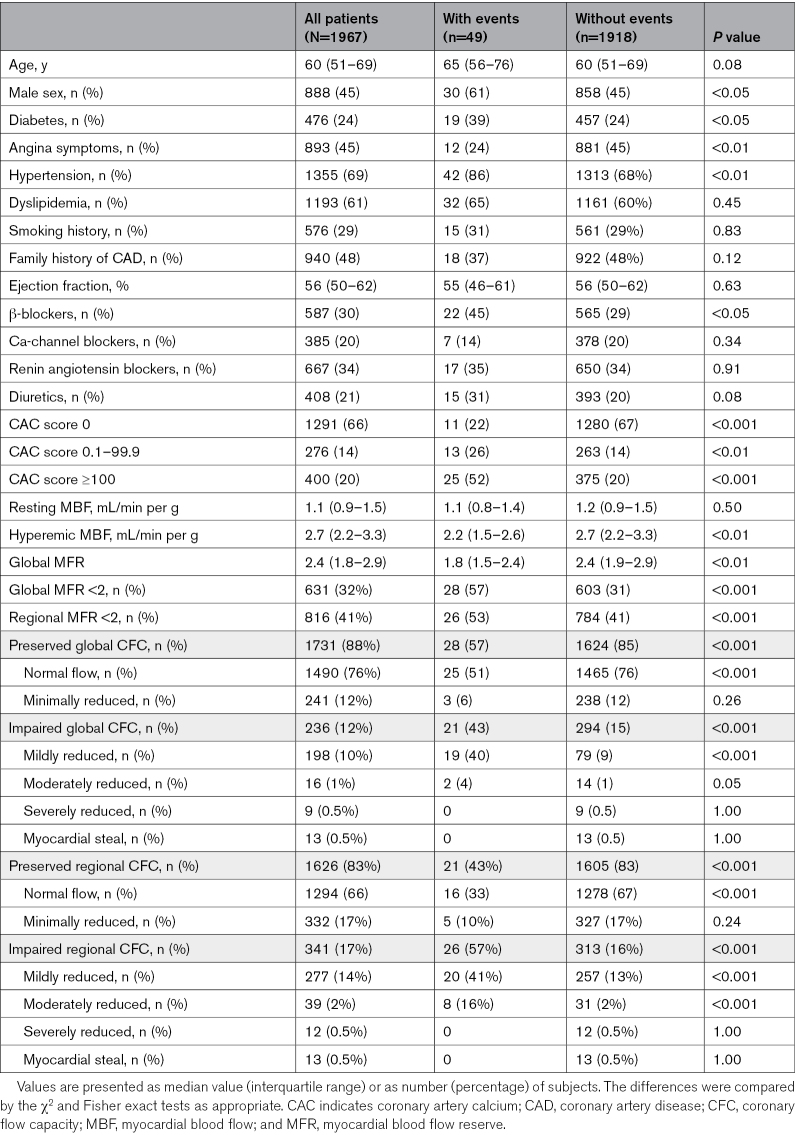
Clinical Characteristics and Imaging Findings According to Events

### Prognostic Value of PET/CT Findings

**Figure 2.**
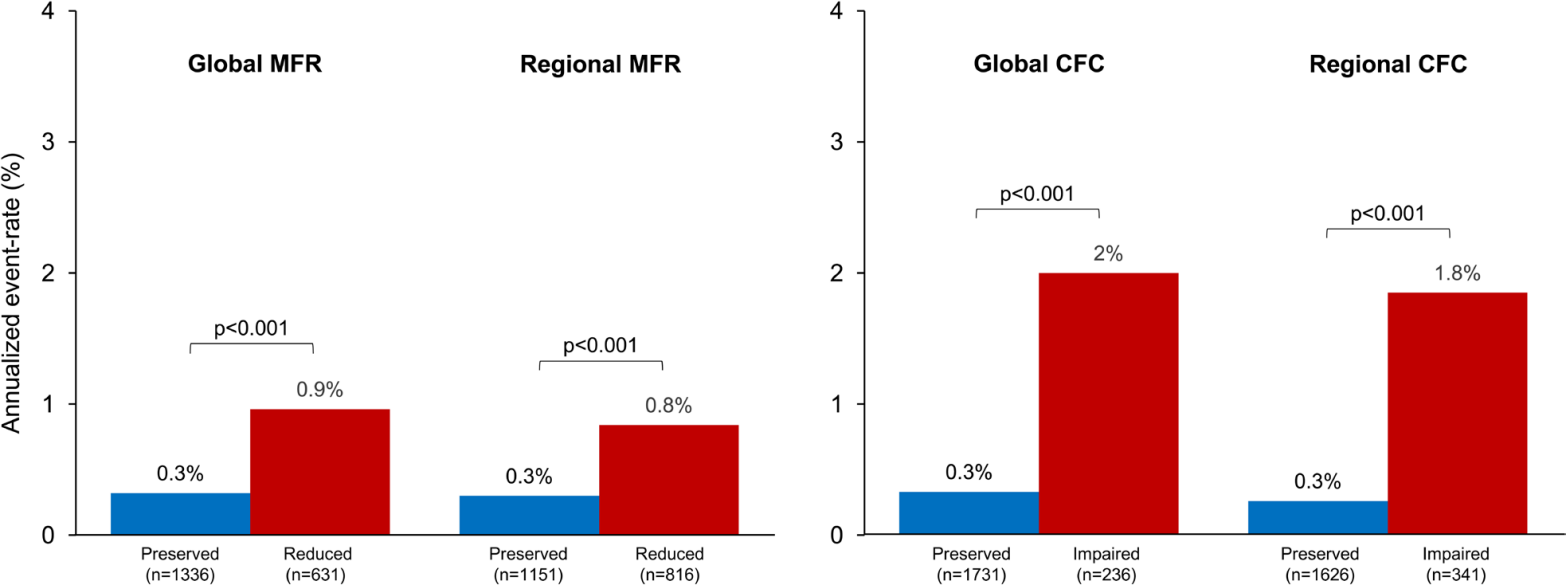
**Annualized event rate (AER) according to myocardial flow reserve (MFR) and coronary flow capacity (CFC).** The AER was higher in patients with reduced MFR and impaired CFC compared with those with preserved MFR and preserved CFC.

**Figure 3.**
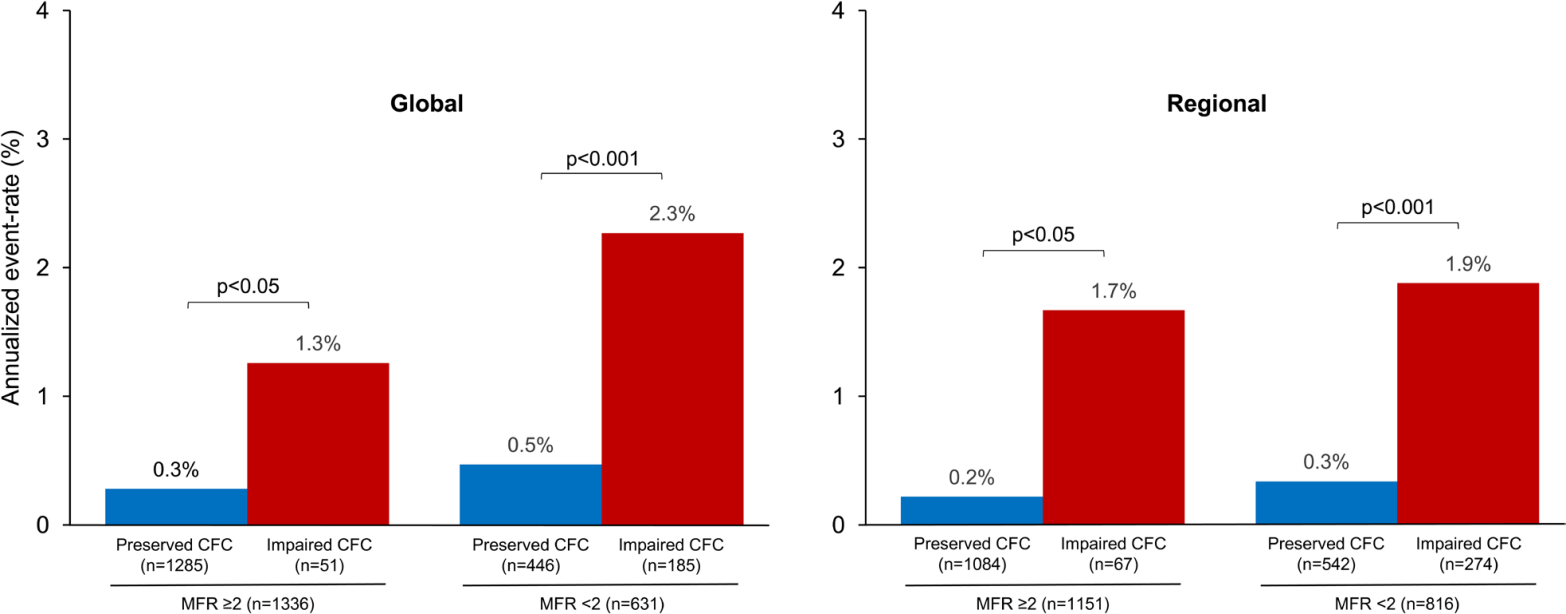
**Annualized event rate (AER) according to coronary flow capacity (CFC) findings in patients with preserved and reduced myocardial flow reserve (MFR).** An impairment of CFC was associated with higher AER independently of MFR findings.

Figure 2 illustrates the rate of cardiac events according to MFR and CFC. The AER was higher in patients with reduced MFR and impaired CFC compared with those with preserved MFR and preserved CFC (all *P*<0.001). Figure 3 shows the AER according to CFC findings in patients with preserved and reduced MFR. Among 1336 patients with preserved global MFR, global CFC was preserved in 1285 (96%) and impaired in the remaining 51 (4%) patients. In the 631 patients with reduced global MFR, global CFC was impaired in 185 (29%) and preserved in 446 (71%) patients. Among 1151 patients with preserved regional MFR, regional CFC was preserved in 1084 (94%) and impaired in the remaining 67 (6%) patients. Finally, in the 816 patients with reduced regional MFR, regional CFC was impaired in 274 (34%) and preserved in 542 (66%) patients.

### Predictors of Outcome

Table 2 shows univariable and multivariable Cox regression analyses in predicting adverse cardiac events. At univariable analysis, age, male sex, diabetes, hypertension, CAC score, global and regional MFR, and CFC were predictors of adverse cardiac events. At multivariable analysis, CAC score and regional CFC resulted in independent predictors of events. At incremental analysis (Table 3), the addition of global MFR to model 1 increased the global χ^2^ from 53.3 to 60.2 (*P*<0.05), while the addition of regional MFR to model 2 did not increase the prediction power. The addition of global CFC to model 3 increased the global χ^2^ to 88.8 (*P*<0.001). The addition of regional CFC to model 4 further increased the global χ^2^ to 99.2 (*P*<0.001).

**Table 2.**
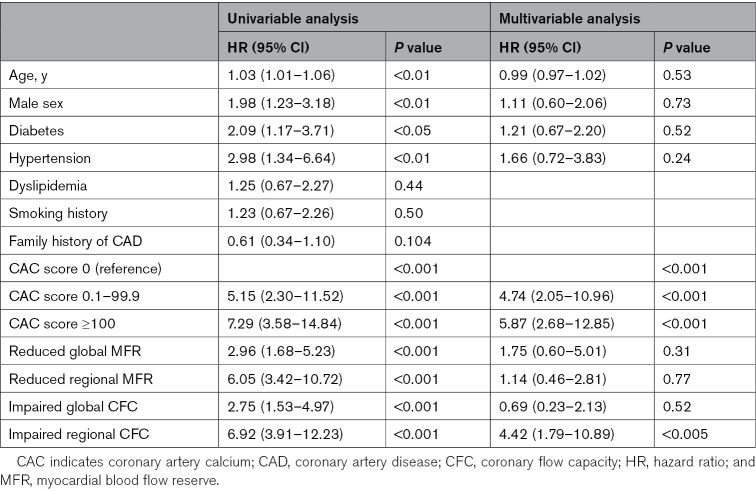
Univariable and Multivariable Cox Regression Analyses in Predicting Adverse Cardiac Events

**Table 3.**
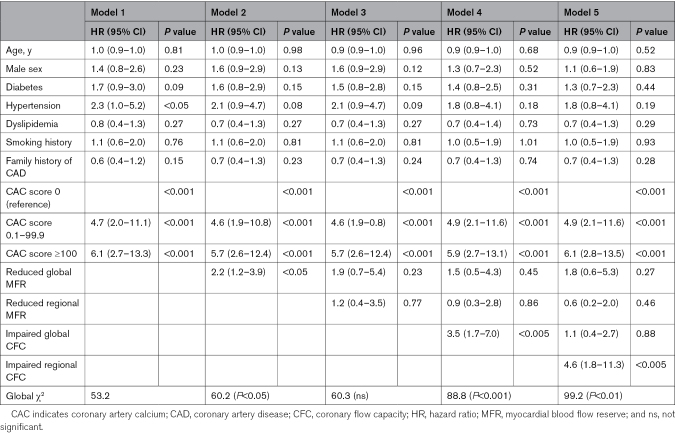
Incremental Analysis for Predicting Adverse Cardiac Events Considering Five Different Models

### Prognostic Evaluation of Combined PET/CT Findings

**Figure 4.**
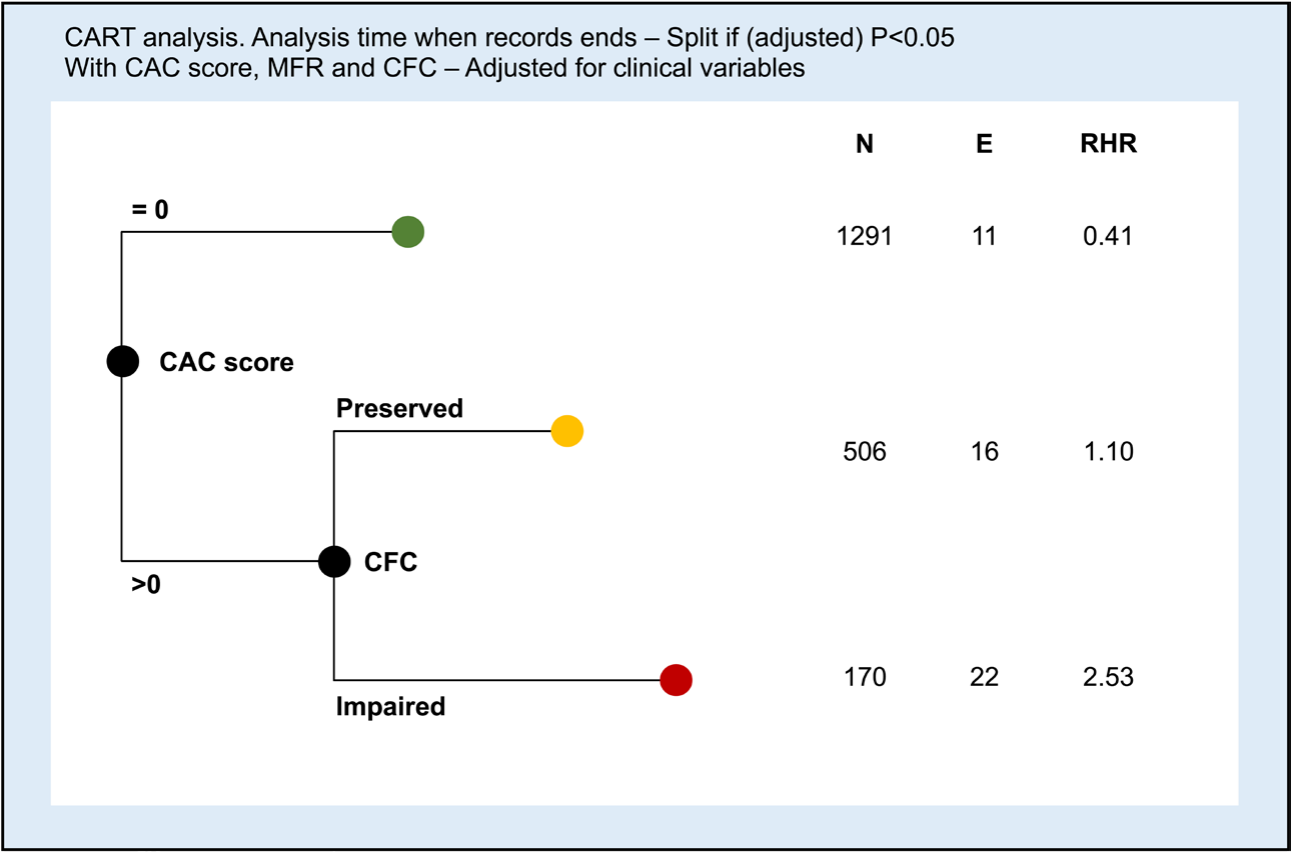
**Classification and regression tree (CART) analysis considering clinical variables, coronary artery calcium score (CAC), regional myocardial flow reserve (MFR), and regional coronary flow capacity (CFC).** The initial split was based on a CAC score of 0. Patients with CAC score >0 were further stratified by CFC. Clinical variables and MFR did not add to the CART structure. E indicates number of events; N, number of patients; and RHR, relative hazard rate.

**Figure 5.**
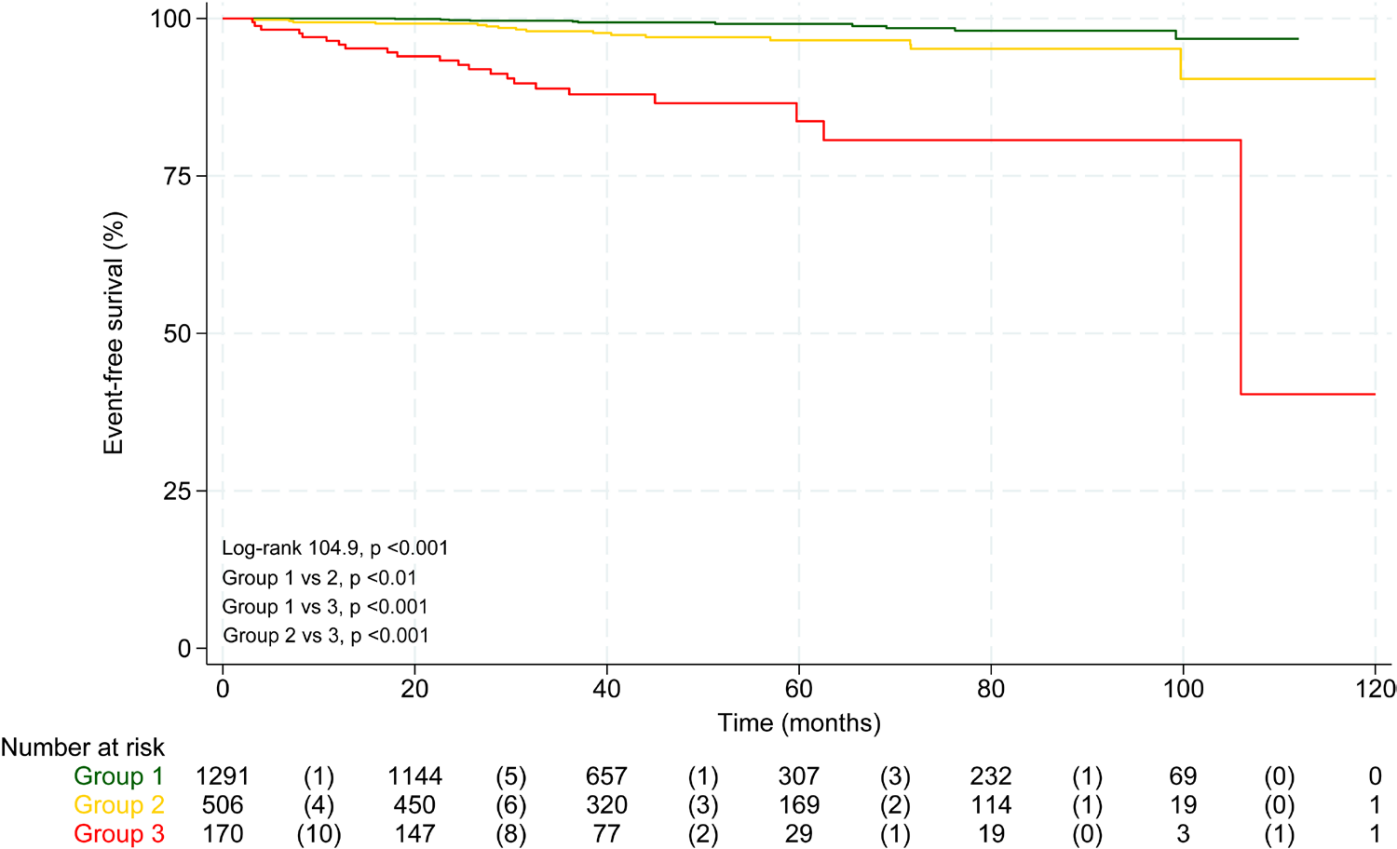
**Event-free survival curves by Kaplan-Meyer analysis according to classification and regression tree analysis.** Survival curves in patients with coronary artery calcium (CAC) score =0 (group 1), patients with CAC score >0, and preserved coronary flow capacity (CFC; group 2), and patients with CAC score >0 and impaired CFC (group 3).

CART analysis (Figure 4) produced 3 terminal groups based on CAC score and regional CFC, while clinical variables and regional MFR did not add to the CART structure. The initial split was on the CAC score, followed by CFC. For patients with CAC score =0 (group 1), no further split was performed, while patients with CAC score >0 were further stratified by preserved (group 2) and impaired (group 3) CFC. The AER (% person-years) was 0.18 (95% CI, 0.09–0.32) for group 1, 0.59 (95% CI, 0.36–0.97) for group 2, and 3.05 (95% CI, 2.01–4.64) for group 3 (*P*_trend_<0.001). The event-free survival curves according to CART groups are reported in Figure 5. There was a significant difference between groups (*P*<0.001).

## Discussion

To our knowledge, this is the first study exploring the prognostic value of CFC derived from ^82^Rb cardiac PET in predicting outcomes in a large series of patients with suspected CAD and normal myocardial perfusion at semiquantitative imaging analysis. From our data, it emerged that, in patients with unknown CAD, the presence of impaired CFC increases the risk of cardiac events compared with patients without such impairment. Moreover, regional CFC provides a more accurate risk stratification compared with global CFC and MFR alone.

Accurate risk stratification has become increasingly important in patients with suspected CAD to adopt appropriate treatment strategies to improve patient outcomes. It should be considered that CAD is a heterogeneous process that may involve myocardial vascular beds at different levels, and its dynamic nature may lead to a long latent phase in which the disease evolves without significant clinical evidence. During this time, the patients can be still asymptomatic or showing normal diagnostic tests.^2^ Radionuclide myocardial perfusion imaging is widely performed in patients with suspected CAD, and it can accurately identify patients at higher risk of future cardiac events.^23–27^ Cardiac imaging by PET/CT has the main advantage of providing accurate measurements of coronary vascular function, in addition to the evaluation of myocardial ischemia.^3^ This aspect is useful in patients with normal perfusion, where the absence of perfusion abnormalities may not exclude the presence of underlying disease,^28^ helping in the identification of microvascular involvement.

Coronary atherosclerotic burden by CAC score evaluation is a strong predictor of cardiac events. In particular, it has been demonstrated a low prevalence of cardiovascular events in patients with a CAC score of 0 and a progressively increased prevalence of events with the increase of CAC score values.^25^ Nowadays, MFR is considered the most validated index of coronary vascular function, and a reduced MFR can be related to the presence of epicardial stenoses and microvascular impairment.^4,5^ The prognostic value of MFR has been extensively investigated, and the presence of impaired MFR is strongly associated with adverse outcomes also in the absence of other perfusion and structural abnormalities.^6–10^ Accordingly, the use of PET/CT allowing to evaluate myocardial perfusion in combination with functional and structural abnormalities demonstrated an accurate risk stratification in patients with low-intermediate risk of CAD.^3^

It should be considered that MFR is a ratio between hyperemic and resting MBFs, and for most patients, hyperemic MBF and MFR findings are concordant in both normal and abnormal results. However, some physiological conditions may affect MFR quantification, producing discordances with hyperemic MBF values that should be carefully interpreted. Therefore, it has been suggested that both parameters, hyperemic MBF and MFR, should be considered in interpreting and reporting test results. In a large cohort of 4029 patients with stable CAD, Gupta et al^29^ tested the ability of hyperemic MBF and MFR, alone or combined, in predicting cardiovascular mortality. The authors identified 4 patients’ categories according to concordant or discordant hyperemic MBF and MFR findings. The category of patients with concordant impairment of both hyperemic MBF and MFR showed the worst prognosis. The authors confirmed that despite that MFR remains a strong predictor of outcome, the integrated evaluation of hyperemic MBF and MFR was helpful in identifying different phenotypes of disease. Fukushima et al^30^ found similar results in 224 patients during a short-term follow-up of 362±277 days.

More recently, the concept of CFC has been introduced as a comprehensive framework for coronary physiology evaluation to overcome some limitations related to using hyperemic MBF or MFR alone.^12,13^ van de Hoef et al,^12^ Johnson and Gould,^13^ and Gould et al^14^ first identified MBF and MFR thresholds under physiological and pathological conditions and integrated these measures in a color-coded scatterplot. Accordingly, the evaluation of CFC has been tested for diagnostic and prognostic purposes.^15^ In particular, CFC was able to provide an accurate quantification of CAD severity resulting as a strong predictor of outcome in 3774 patients with both suspected and known CADs.^14^

Dietz et al^31^ compared hyperemic MBF, global MFR, and CFC, obtained by cardiac PET, in predicting short-term outcomes in a small patient population of 234 patients with suspected myocardial ischemia using the latest silicon photomultiplier PET technology with low-dose ^82^Rb imaging. They found that despite that all PET parameters are powerful predictors of cardiovascular events, only reduced hyperemic MBF resulted in being independently associated with outcome.^31^ Miura et al^17^ proposed a more practical approach, introducing the concept of modified CFC, which integrates regional MFR and hyperemic MBF values into the definition of normal or abnormal CFC. This approach has been tested in a limited number of patients (n=137) without evidence of obstructive CAD at coronary angiography to identify the presence of microvascular dysfunction and its prognostic implications.^17^ The authors found that 25% of patients had impairment of CFC and showed an increased risk of cardiovascular mortality. More recently, de Winter et al^18^ tested the prognostic impact of CFC by ^15^O-H_2_O PET imaging in 1300 patients with both suspected and known CADs. They found that CFC was an independent predictor of outcome also after adjusting for clinical variables.

In our study, we aimed to test the prognostic value of CFC in a large series of patients without evidence of previous CAD and normal myocardial perfusion at semiquantitative imaging analysis, testing the role of this feature in combination with other parameters obtained by ^82^Rb PET/CT. From our data, it emerged that AER, at both global and regional analyses, was significantly higher in patients with impaired CFC compared with those with preserved CFC and in patients with reduced MFR compared with those with preserved MFR. Interestingly, the large majority of the patients with normal MFR had also normal CFC. On the contrary, a low percentage of patients with reduced MFR had abnormal CFC. In particular, the risk of cardiac events resulted to be significantly higher in the presence of impaired CFC also in patients with normal MFR, at both global and regional analyses, suggesting that CFC is a powerful marker of coronary vascular status able to improve risk stratification also in patients with preserved MFR.

However, the CAC score and both global and regional CFCs resulted as independent predictors of events at multivariate analysis, with a higher hazard ratio value for regional CFC compared with global CFC. Indeed, the results of CART analysis clearly indicate that a significant increase in hazard ratio becomes apparent only for impairment of regional CFC in patients presenting a CAC score >0. From this study, it has clearly emerged that regional CFC alone may be sufficient to separate the high-risk versus low-risk patients without the intermediate step of global or regional MFR.

### Limitations

This is an observational study, and CFC values were retrospectively collected. Moreover, despite that CFC is able to identify a group of patients at higher risk of cardiac events, the clinical impact of such identification cannot be provided. Further studies are requested to answer this specific question.

### Conclusions

In patients with suspected CAD and normal myocardial perfusion at semiquantitative imaging analysis, CFC derived from ^82^Rb cardiac PET imaging is able to identify patients at higher risk of cardiovascular events. In particular, in patients with both normal and reduced MFRs, the presence of impaired CFC helps to better identify patients at risk of event. The evaluation of CFC shows a higher prognostic impact compared with MFR alone.

## ARTICLE INFORMATION

### Sources of Funding

None.

### Disclosures

None.
